# The fundamental association between mental health and life satisfaction: results from successive waves of a Canadian national survey

**DOI:** 10.1186/s12889-018-5235-x

**Published:** 2018-03-12

**Authors:** Patrick Lombardo, Wayne Jones, Liangliang Wang, Xin Shen, Elliot M. Goldner

**Affiliations:** 1 Centre for Applied Research in Mental Health & Addiction (CARMHA), Simon Fraser University, Faculty of Health Sciences, Suite 2400 – 515 West Hastings St, Vancouver, BC V6B 5K3 Canada; 2 Department of Statistics and Actuarial Science, Simon Fraser University, Vancouver, Canada; 3 College of Economics and Management, Shanghai Ocean University, Shanghai, China

**Keywords:** Life satisfaction, Subjective well-being, Quality of life, Happiness, Positive mental health, Mental health, Income

## Abstract

**Background:**

A self-reported life satisfaction question is routinely used as an indicator of societal well-being. Several studies support that mental illness is an important determinant for life satisfaction and improvement of mental healthcare access therefore could have beneficial effects on a population’s life satisfaction. However, only a few studies report the relationship between subjective mental health and life satisfaction. Subjective mental health is a broader concept than the presence or absence of psychopathology. In this study, we examine the strength of the association between a self-reported mental health question and self-reported life satisfaction, taking into account other relevant factors.

**Methods:**

We conducted this analysis using successive waves of the Canadian Community Health Survey (CCHS) collected between 2003 and 2012. Respondents included more than 400,000 participants aged 12 and over. We extracted information on self-reported mental health, socio-demographic and other factors and examined correlation with self-reported life satisfaction using a proportional ordered logistic regression.

**Results:**

Life satisfaction was strongly associated with self-reported mental health, even after simultaneously considering factors such as income, general health, and gender. The poor-self-reported mental health group had a particularly low life satisfaction. In the fair-self-reported mental health category, the odds of having a higher life satisfaction were 2.35 (95% CI 2.21 to 2.50) times higher than the odds in the poor category. In contrast, for the “between 60,000 CAD and 79,999 CAD” household income category, the odds of having a higher life satisfaction were only 1.96 (95% CI 1.90 to 2.01) times higher than the odds in the “less than 19,999 CAD” category.

**Conclusions:**

Subjective mental health contributes highly to life satisfaction, being more strongly associated than other selected previously known factors. Future studies could be useful to deepen our understanding of the interplay between subjective mental health, mental illness and life satisfaction. This may be beneficial for developing public health policies that optimize mental health promotion, illness prevention and treatment of mental disorders to enhance life satisfaction in the general population.

**Electronic supplementary material:**

The online version of this article (10.1186/s12889-018-5235-x) contains supplementary material, which is available to authorized users.

## Background

A landmark paper by Prince and colleagues [1] surmised that there is “no health without mental health”; poor mental health is a risk factor for multiple diseases, a comorbidity of many physical health problems and complicates the care of co-occurring diseases. The World Health Organization (WHO) recognizes the essential role of mental health for achieving overall health and mental health has been incorporated into population health strategies by various jurisdictions [2], including the United Kingdom’s Department of Health’s “no health without mental health” strategy [3].

Measurement of subjective well-being at the population level is a well-established discipline of economics and sociology. Interest in subjective well-being grew after the late-2000s financial crisis, when reforms to the financial system were proposed [4, 5]. A key index of well-being and the societal progress of a population is the self-reported measure of life satisfaction. The annual World Happiness Report [5] provides self-reported mean life satisfaction scores of more than 150 countries to reflect social progress. Population-level life satisfaction scores are widely reported, and studies support their validity [6, 7]. Research on the determinants of subjective well-being has shown that health status is one of the most important factors associated with subjective well-being [8–10]. Moreover, a strong and robust inverse association exists between life satisfaction and the presence of mental illness [11–17].

Mental health is a complex concept that goes beyond the mere absence of mental illness and WHO defines it as, a “state of well-being in which an individual realizes his or her own abilities, can cope with the normal stresses of life, can work productively and is able to make a contribution to his or her community” [18]. Indeed, mental health and mental illness are best conceptualized as two dimensions that are strongly correlated but that are not at opposite ends of the same scale: there are people that have low levels of mental health who do not suffer from a psychopathology [19–21].

Rather than defining mental health according to the presence or absence of mental illness, the current study investigates the relationship between life satisfaction and subjective mental health, measured by self-reported questions. To our knowledge, only a few studies have examined this relationship [22–24]. In 2010, Sharpe et al. [22] published a report that explored the geographical differences in life satisfaction using the Canadian Community Health Survey (CCHS) of 2007–2008. To characterize the population within each geographical area they explored the relationship between life satisfaction and different factors and found that self-reported mental health was strongly associated with life satisfaction.

This study explores the relationship between self-reported mental health and life satisfaction over time, assessing successive waves of the CCHS comprising more than 600,000 participants from 2003 to 2012. It also quantifies the relative contribution of mental health to life satisfaction relative to other factors.

## Methods

### Database

From 2001, Statistics Canada collected data on health status, health utilization and health determinants of the Canadian population aged 12 and over for the Canadian Community Health Survey (CCHS). The respondents selected for this cross-sectional survey were a representative sample of 98% of the Canadian population. About 130,000 respondents every 2 years from 2001 to 2005 and 65,000 respondents annually from 2007 were included. The survey collected responses from participants using a computer-assisted application, by phone or by face-to-face interviews. The survey excluded individuals living on Indian Reserves and on Crown Lands, institutional residents, full-time members of the Canadian Forces and residents of certain remote regions.

The survey determined geographical selection of respondents by an approach that allocates a minimal number of respondents for each Health Area, while providing a number of respondents for each province proportional to its population. To ensure representability, geographical and socio-economic characteristics collected from other surveys were also considered. The survey contacts one person per household and participation is voluntary. The response rate varied from 67 (in CCHS 2011–2012) to 80.7% (CCHS 2003).

Public Use Microdata Files (PUMF) derived from the master files to ensure respondent security were available from 2003 to 2012. In the PUMF files, variables that could lead to identification of an individual were deleted or transformed into broader categories. Additional information regarding the CCHS surveys can be found on the Statistics Canada website [25].

In this study, we used five CCHS surveys: CCHS 2003, CCHS 2005, CCHS 2007–2008, CCHS 2009–2010, CCHS 2011–2012.

### Survey

After obtaining demographic information from the respondents, the CCHS survey opens with the following statement: *“This survey deals with various aspects of your health. I will be asking about such things as physical activity, social relationships, and health status. By health, we mean not only the absence of disease or injury but also physical, mental and social well-being.”* Table 1 lists the factors and their corresponding categories extracted from the CCHS surveys used in the current study.

**Table 1:** Factors selected for analysis

Factors	Categories
Life satisfaction	5 categories from “very dissatisfied” to “very satisfied.” From 2009, the scale was changed to 0 to 10.
Age category	15 categories: 12–14, 15–19, …, 75–79, 80 and over.
Gender	Male or Female
Province or territory of residence	11 categories: 10 provinces and a category combining the 3 territories.
Year of inclusion	5 categories: 2003, 2005, 2007–2008, 2009–2010, and 2011–2012
Self-reported mental health	5 categories from “poor” to “excellent.”
Self-reported general Health	5 categories from “poor” to “excellent.”
Amount of stress in life	5 categories from “not at all” to “extreme.”
Sense of community belonging	4 categories from “very weak” to “very strong.”
Household income	5 categories. Category cut-offs changed over time (see text).
Personal income	6 categories. “No income” and 5 other categories identical to household income.
Self-reported mood or anxiety disorders	Yes, No.

The survey questions are in the same order from year to year. The first question addresses general health followed by a question on the variation of health compared to the previous year. The survey then asks questions about life satisfaction and mental health. Details are in Table 2.

**Table 2:** Life satisfaction and self-reported mental health items

Life satisfaction item
How satisfied are you with your life in general?
1. Very satisfied 2. Satisfied 3. Neither satisfied nor dissatisfied 4. Dissatisfied 5. Very dissatisfied
Self-reported mental health item
In general, would you say your mental health is:
1. Excellent? 2. Very good? 3. Good? 4. Fair? 5. Poor?

From 2009 onwards, the life satisfaction question changed measure to a scale from 0 to 10: *“Using a scale of 0 to 10, where 0 means “Very dissatisfied” and 10 means “Very satisfied,” how do you feel about your life as a whole right now?”* We extracted self-reported mood or anxiety disorders as a binary variable from a series of questions related to chronic conditions. Household income is a variable with five categories going from the lowest to the highest income. Personal income had a supplementary category that grouped respondents without personal income. Before 2007, the cut-offs between annual income categories were set at $15,000, $30,000, $50,000 and $80,000 CAD. From 2007, these cut-offs changed to $20,000, $40,000, $60,000, and $80,000 CAD respectively. From 2011, Statistics Canada used an imputation method to predict missing household data using a regression model including respondent and household members’ characteristics and adjusted using the median household income for each postal code by household size obtained from a tax database.

### Statistical analysis

To perform this analysis, we pooled data from the five CCHS (from 2003 to 2012) surveys and the year of inclusion was treated as a factor. In the preliminary analysis, the life satisfaction question was considered as a score from 1 to 5, where 1 corresponded to “very dissatisfied” and 5 to “very satisfied” and the different categories for each factor (listed in Table 1) were tested using unpaired t-tests without adjustment. The life satisfaction scale from 0 to 10, used from 2009, was converted to five categories using cut-off points that reproduced the distribution found with the categorical life satisfaction question before 2009.

In order to determine the association of each factor with life satisfaction while adjusting all other variables, we analyzed all variables using an ordinal logistic regression—proportional odds model (cumulative logit model). Different from linear regression, this method has the advantage of treating the outcome variable, life satisfaction, as ordinal, and not as a continuous variable. We tested the proportional odds assumption for each variable. A partial proportional odds model could be used if this assumption was severely violated. Otherwise, the proportional odds model was preferable for simplicity of interpretation and computation. To explore the issue of potential collinearity, we tested the strength of the association between all variables used in the analysis using Godman and Kruskal’s gamma.

The BIC method penalizes logistic regression with an increased number of parameters that has a small effect on the explained variable and allows selecting only factors that have an impact on the explained variable.

To check that the results we obtained are not dependent on how life satisfaction is scaled, and to decrease the amount of missing data, a sensitivity analysis was performed using CCHS 2011–2012 only, using the same procedure, except that the life satisfaction question was now scaled from 0 to 10 and the personal income factor was removed. This allowed us to confirm results with a life-satisfaction question scale of 0 to 10, in a survey with a lower quantity of missing data. Indeed, for the CCHS 2011–2012, Statistics Canada imputed the household income variable (more details above).

We performed analyses using R version 3.3.2 [26] with “car,” “ggplot2” [27], “glmulti” [28], “plyr”, “MASS”, and “VGAM” [29] packages.

## Results

### Participants characteristics and missing data

Data obtained from the different surveys pooled together from 2003 to 2012 included responses from 646,471 participants collected by the CCHS. For each period, about 129,000 participants were interviewed (the lowest was 124,929 in 2011–2012, and the highest 134,072 in 2003). Data about household income and personal income were missing in 13.1% and 19.9% of the participants, respectively, and in the group of participants with missing data, household income was missing from 55.0% of the respondents and personal income from 83.1% of the respondents. For the other variables, the proportion of missing data was 3.5% maximum. Data were mainly missing from participants under 19 (63.4%) and over 65 years-old (27.6%). Although data were complete for all the selected variables in 491,850 participants (76.1%), the highest (over 80 years) and lowest (12–15 years) age categories had to be excluded in the cumulative logit model analysis because of the small number of participants in these categories. Therefore, 466,574 (72.1%) participants were included in the cumulative logit analysis. Included respondents were similar regarding gender (54.2% of women in the group included vs 54.4% overall) and other variables.

### Overview of survey responses

**Fig. 1:**
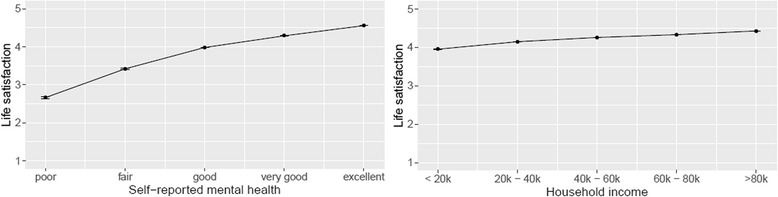
Mean and 95% confidence interval for life satisfaction by household income in CAD & self-reported mental health

91.2% of the participants rated life satisfaction as “satisfied” or “very satisfied” and 71.8% rated their mental health as “very good” or “excellent.” The overall life satisfaction mean score was of 4.26 (standard deviation = 0.73, maximum score was 5). People with poor or fair self-reported mental health had a particularly low life satisfaction. We show the scores for each factor category in Table 3. The “age” and the “province or territory of residence” categories are shown in the Additional file 1: Tables S1 and S2. In the age variable, the life satisfaction score ranged from 4.18 (95% CI 4.18 to 4.19, *p*-value < 0.0001) in the 50–54 year-old category, to 4.45 (95% CI 4.44 to 4.46, *p*-value < 0.0001) in the 12–14 year-old category. The province of British Columbia had the lowest life satisfaction score (4.23, 95% CI 4.22 to 4.23, *p*-value < 0.0001), while the province of Prince Edward Island had the highest (4.32 95% CI 4.30 to 4.33, *p*-value < 0.0001). When household income increased, life satisfaction increased, but to a lesser extent than with increased self-reported mental health (Fig. 1). The life satisfaction score did not increase linearly with personal income. Compared to the other factors, the life satisfaction score remained stable for the CCHS inclusion year. According to the Godman and Kruskal’s gamma testing the association between variables, multicollinearity should not affect our results.

**Table 3:** Life satisfaction average score and 95% confidence interval for each category

Self-reported mental health	poor	fair	good	Very good	excellent	
	2.66 (2.63 to 2.69)	3.42 (3.41 to 3.43)	3.98 (3.98 to 3.98)	4.29 (4.28 to 4.29)	4.55 (4.55 to 4.56)	
Self-reported general health	poor	fair	good	Very good	excellent	
	3.25 (3.24 to 3.27)	3.83 (3.83 to 3.84)	4.14 (4.14 to 4.15)	4.39 (4.38 to 4.39)	4.57 (4.57 to 4.57)	
Amount of stress in life	Extreme	Quite a bit	A bit	Not Very	Not at all	
	3.59 (3.57 to 3.61)	4.00 (3.99 to 4.00)	4.23 (4.23 to 4.24)	4.39 (4.39 to 4.40)	4.51 (4.51 to 4.52)	
Sense of community belonging	Very weak	Some weak	Some strong	Very strong		
	3.90 (3.89 to 3.90)	4.12 (4.11 to 4.12)	4.31 (4.31 to 4.31)	4.45 (4.45 to 4.46)		
Household income (in CAD)	< 19,999	20,000–39,999	40,000–59,999	60,000–79,999	> 80,000	
	3.95 (3.94 to 3.96)	4.14 (4.14 to 4.15)	4.25 (4.25 to 4.26) p-value: 0.222	4.33 (4.32 to 4.33)	4.42 (4.42 to 4.43)	
Change in health compared to 1 year ago	Much worse	Some worse	Same	Some Better	Much better	
	3.34 (3.32 to 3.37)	3.93 (3.93 to 3.94)	4.31 (4.31 to 4.31)	4.25 (4.24 to 4.25)	4.40 (4.39 to 4–41)	
Self-reported mood or anxiety disorder	Yes	No				
	3.78 (3.78 to 3.79)	4.31 (4.31 to 4.31)				
Personal income (in CAD)	No income	< 19,999	20,000–39,999	40,000–59,999	60,000–79,999	> 80,000
	4.26 (4.25 to 4.27) p-value:0.2256	4.13 (4.12 to 4.13)	4.22 (4.22 to 4.23)	4.31 (4.30 to 4.31)	4.37 (4.36 to 4.37)	4.43 (4.42 to 4.43)
Year of inclusion	2003	2005	2007–2008	2009–2010	2011–2012	
	4.24 (4.24 to 4.25)	4.25 (4.25 to 4.26) *p*-value: 0.220	4.26 (4.26 to 4.27) *p*-value: 0.052	4.26 (4.26 to 4.26) *p*-value: 0.245	4.27 (4.26 to 4.27)	

Unless otherwise specified, *p*-values of the displayed means are < 0.0001

### The year of inclusion does not influence life satisfaction

All the variables were analyzed using ordinal logistic regression: all possible multiple ordinal logistic regressions were generated and the proportional odds model with the lowest Bayesian information criterion (BIC) selected. The ordinal regression model with the lowest BIC included all the factors except the year of inclusion. This demonstrated that the year of inclusion did not have an important impact on the life satisfaction responses. In other words, the year of inclusion factor did not influence the correlations between life satisfaction and the other parameters.

### Self-reported mental health is strongly associated with life satisfaction

In order to measure the effect of each factor considering all other factors, the cumulative odds ratio was calculated for each factor within the selected logistic regression with a cumulative logit model. The most important factors, with at least one odds ratio exceeding 1.5, are displayed in Table 4. The complete Table is available in the Additional file 1: Table S3. Although some factors, such as “age category” and “household income” violated the proportional assumption of the proportional odds model, we presented the results from the proportional odds model for simplicity. As example of how to interpret the odds ratios obtained with our model shown in Table 4, consider the odds ratio of “fair” self-reported mental health of 2.35 (95% CI 2.21 to 2.50). This means that if the “self-reported mental health” increases from “poor” (which is the reference group) to “fair”, the odds of life satisfaction being higher than one category versus being equal to or lower than this category is 2.35. Thus, compared to all other factors, self-reported mental health had a strong association with life satisfaction. By comparison, the odds ratio of the “fair” self-reported mental health in the example above, of 2.35, is higher than the odds ratio obtained for the “between 60,000 CAD and 79,999 CAD” household income category (1.96; 95% CI 1.90 to 2.01, *p*-value < 0.0001).

**Table 4:** Odds ratios of the most important factors associated with life satisfaction

	Odds ratio (adjusted for the other factors)	95% confidence interval
Self-reported mental health		
Poor	Ref	
Fair	2.35	2.21 to 2.50
Good	5.89	5.54 to 6.22
Very good	11.60	10.91 to 12.34
Excellent	25.65	24.12 to 27.29
Self-reported general health		
Poor	Ref	
Fair	2.08	1.99 to 2.16
Good	3.16	3.03 to 3.29
Very good	4.92	4.73 to 5.13
Excellent	7.33	7.03 to 7.65
Amount of stress in life		
Extreme	Ref	
Quite a bit	1.48	1.43 to 1.54
A bit	2.15	2.07 to 2.23
Not Very	3.03	2.92 to 3.14
Not at all	4.68	4.50 to 4.87
Sense of community belonging		
Very weak	Ref	
Some Weak	1.22	1.19 to 1.25
Some Strong	1.73	1.69 to 1.77
Very Strong	2.64	2.57 to 2.71
Household income		
Less than 19,999 CAD	Ref	
Between 20,000 CAD and 39,999 CAD	1.29	1.26 to 1.33
Between 40,000 CAD and 59,999 CAD	1.63	1.58 to 1.67
Between 60,000 CAD and 79,999 CAD	1.96	1.90 to 2.01
More than 80,000 CAD	2.53	2.46 to 2.60
Change in health compared to 1 year ago		
Much worse	Ref	
Some worse	1.38	1.31 to 1.46
The same	1.71	1.62 to 1.81
Some better	1.77	1.67 to 1.87
Much better	2.35	2.23 to 2.49

All *p*-values of the displayed factors are < 0.0001

Factors with smaller odds ratios are not displayed

Self-reported general health and self-reported mental health have the same category divisions, but self-reported mental health was associated with higher odds of life satisfaction. Differences in stress and sense of community belonging were associated with changes in life satisfaction that were notably important if compared to household income.

### The importance of self-reported mental health is confirmed by a sensitivity analysis

Finally, we performed a sensitivity analysis, using the CCHS 2011–2012 survey only. In this survey, life satisfaction was scored from 0 to 10 and household income was inferred by Statistics Canada. In the analysis, data were complete for 94.3% of the respondents. Cumulative odds ratios obtained with the cumulative logit model were similar to the original analysis, with the odds ratio of the “fair” self-reported mental health of 2.61 (95% CI 2.32 to 2.93), and 1.42 (95% CI 1.36 to 1.48) for the group “between 60,000 CAD and 79,999 CAD”. The complete results are available in the Additional file 1: Table S4.

## Discussion

This study found that the subjective mental health status was highly associated with life satisfaction compared to using a cumulative logit model used to isolate the effect of each factor from the others. People with poor self-rated mental health had a particularly low life satisfaction. The difference in odds ratios of having a higher life satisfaction between the “poor” and the “fair” subjective mental health was higher than the difference between the “less than 19,999 CAD” and the “between 60,000 CAD and 79,999 CAD” household income categories. The mood or anxiety disorder status odds ratio was relatively small, which may suggest that the subjective evaluation of the mental health status encompassed the actual mood or anxiety disorder status. In other words, an individual suffering from mood or anxiety disorder would very often report low mental health status, but there are other parameters outside of the mood and anxiety status that have an impact on the subjective mental health status.

Our study does not indicate that self-reported general health includes self-reported mental health. If this was the case, the odds ratio for the self-reported mental health factor in the cumulative logit model would be very low. In concordance with a previous study on the relationship between the self-reported health question and different health scales, the authors hypothesized that the self-reported health question reflects rather the physical than the mental health status [30].

The life satisfaction score appeared stable through the successive waves of the survey from 2003 to 2012. Moreover, the exploration of the multiple ordered logistic regressions using the BIC showed that the “year of inclusion” parameter was the weakest factor and therefore not part of the best model. This suggests that life satisfaction was less related to the year of inclusion than to the other factors selected in our study and implies that social or economic events happening over this timeframe had a smaller impact on Canadians’ life satisfaction than other self-rated factors.

Strengths of our study include its large size, made possible by the multiple CCHS surveys undertaken from 2003 to 2012, in which more than 600,000 interviews were performed. Moreover, we carried out specific analyses that treated the life satisfaction variable as an ordinal variable instead of transforming it to a continuous variable.

Our study has some limitations. First, 23.9% of the participants were excluded from the main analysis because of missing income information. However, the sensitivity analysis performed using CCHS 2011–2012 with complete data for 94.3%, showed similar results. Therefore, the impact of the missing data on the final results may be mitigated. Second, as the CCHS was designed to evaluate health, and the general health question always preceded the life satisfaction question, the health dimension may be over estimated when participants evaluated their life satisfaction [31]. Third, the results did not inform about a causal relationship between life satisfaction and the different factors.

Usually, studies on well-being use different indicators of mental illness when mental health is assessed and all show robust associations between mental illness and life dissatisfaction that persisted after adjustment [11–14]. This association was extended to the national level in three studies with various indicators of mental illness in European countries. Touburg and Veenhoven [16] found that the national life satisfaction mean score was related to the percentage of the health budget spent on mental health in developed nations. Layard et al. [17] assessed the British, Australian and German population surveys and found that mental illness is a key determinant of life dissatisfaction.

Two studies assessed the relationship between subjective mental health and life satisfaction [23, 24]. Both used a “not good days, mental health” question (Now thinking about your mental health, which includes stress, depression and problems with emotions, for how many days during the past 30 days was your mental health not good?). Michalos and Zumbo [23] used six surveys from the United States Center for Disease Control and Prevention and explored the relationship between subjective mental health, physical health, general health and life satisfaction. The subjective mental health variable was more correlated with life satisfaction compared to other variables tested. Zullig et al. [24] found a similar relationship exploring these indicators in 13 to18 year-old adolescents in South Carolina (United-States).

Instead of using the mental illness status, we used the subjective mental health status to define mental health in our study. This question is only moderately correlated with mental illness and refers to other aspects of mental health that are more complex than just the presence or absence of a psychopathology [32, 33]. In line with our results and evidence from previous studies; whether mental health is measured by a self-rated question or by mental illness status, it appears that mental health is a valuable resource for a happy population. This outlines the importance to further our understanding of the relationship between subjective mental health, mental illness and life satisfaction.

## Conclusion

Our study shows that subjective mental health is an important factor associated with life satisfaction and could be a valuable resource for a happy population. However, this aspect of mental health tended to be ignored given the dominant approach that defines mental health as the absence or presence of a psychopathology [11–17]. Owing to the importance of subjective mental health for life satisfaction reported by our study, further research may be useful to understand the relationship between subjective mental health, mental illness and well-being. This may help to enhance public health strategies, including mental health promotion, mental illness prevention and treatment to promote life satisfaction in the general population.

## Additional file

Additional file 1: **Table S1**. Life satisfaction mean score and 95% confidence interval for each age category. **Table S2**. Life satisfaction mean score and 95% confidence interval for each province or territory category. **Table S3**. Odds ratios of the factors associated with life satisfaction. **Table S4**. Sensitivity analysis: odds ratios of the factors associated with life satisfaction. (DOCX 44 kb)

## Abbreviations

CCHS: Canadian Community Health Survey

## References

[1] Prince M, Patel V, Saxena S, Maj M, Maselko J, Phillips MR (2007). No health without mental health. Lancet.

[2] World Health Organization. Mental health action plan 2013-2020. 2013. http://www.who.int/mental_health/publications/action_plan/en/.

[3] Department of Health. No Health Without Mental Health: A Cross-government Mental Health Outcomes Strategy for People of All Ages. London: Stationery Office; 2011.

[4] Stiglitz J, Sen A, Fitoussi J-P. The Measurement of Economic Performance and Social Progress Revisited. Paris: Commission on the Measurement of Economic Performance and Social Progress; 2009.

[5] Helliwell J, Layard R, Sachs J (2016). World happiness report 2016 update.

[6] Diener E, Inglehart R, Tay L (2012). Theory and validity of life satisfaction scales. Soc Indic Res.

[7] Organisation for Economic Co-operation and Development (OECD). OECD Guidelines on Measuring Subjective Well-being. Paris: OECD Publishing; 2013. 24600748

[8] Boarini R, Comola M, Smith C, Manchin R, de Keulenaer F. What makes for a better life? OECD Publishing; 2012.

[9] Diener E, Chan MY (2011). Happy people live longer: subjective well-being contributes to health and longevity. Appl Psychol Heal Well-Being.

[10] Dolan P, Peasgood T, White M (2008). Do we really know what makes us happy? A review of the economic literature on the factors associated with subjective well-being. J Econ Psychol.

[11] Rissanen T, Viinamäki H, Lehto SM, Hintikka J, Honkalampi K, Saharinen T, et al. The role of mental health, personality disorders and childhood adversities in relation to life satisfaction in a sample of general population. Nord. J. Psychiatry Taylor & Francis. 2013;67:109–15. 10.3109/08039488.2012.687766 22594856

[12] Strine TW, Kroenke K, Dhingra S, Balluz LS, Gonzalez O, Berry JT (2009). The associations between depression, health-related quality of life, social support, life satisfaction, and disability in community-dwelling US adults. J Nerv Ment Dis.

[13] Fergusson DM, McLeod GFH, Horwood LJ, Swain NR, Chapple S, Poulton R (2015). Life satisfaction and mental health problems (18 to 35 years). Psychol Med Cambridge University Press.

[14] Rissanen T, Viinamäki H, Honkalampi K, Lehto SM, Hintikka J, Saharinen T (2011). Long term life dissatisfaction and subsequent major depressive disorder and poor mental health. BMC Psychiatry.

[15] Bray I, Gunnell D (2006). Suicide rates, life satisfaction and happiness as markers for population mental health. Soc Psychiatry Psychiatr Epidemiol.

[16] Touburg G, Veenhoven R (2015). Mental health care and average happiness: strong effect in developed nations. Adm Policy Ment Heal Ment Heal Serv Res Springer US.

[17] Layard R, Chisholm D, Patel V, Saxena S. Mental illness and unhappiness. CEP Discuss. Pap. Centre for Economic Performance, LSE; 2013;

[18] World Health Organization. The World Health Report 2001: mental health new understanding, new hope. Geneva: World Health Organization; 2001.

[19] Westerhof GJ, Keyes CLM (2010). Mental illness and mental health: the two continua model across the lifespan. J Adult Dev.

[20] Keyes CLM (2005). Mental illness and/or mental health? Investigating axioms of the complete state model of health. J Consult Clin Psychol American Psychological Association.

[21] Antaramian SP, Huebner ES, Hills KJ, Valois RF (2010). A dual-factor model of mental health: toward a more comprehensive understanding of youth functioning. Am J Orthopsychiatry Wiley-Blackwell Publishing Ltd.

[22] Sharpe A, Ghanghro A, Johnson E, Kidwai A (2010). Does money matter?: determining the happiness of Canadians.

[23] Michalos AC, Zumbo BD (2002). Healthy days, Health satisfaction and satisfaction with the overall quality of life. Soc Indic Res.

[24] Zullig KJ, Valois RF, Huebner ES, Drane JW (2005). Adolescent health-related quality of life and perceived satisfaction with life. Qual Life Res.

[25] Statistics Canada: Canada’s national statistical agency. http://www.statcan.gc.ca/. Accessed 03 Mar 2017.

[26] R Development Core Team. R: a language and environment for statistical computing. Vienna: the R Foundation for Statistical Computing; 2015.

[27] Valero-Mora PM, Valero-Mora MP. ggplot2: elegant graphics for data analysis. J Stat Softw Foundation for Open Access Statistics. 2010;35:65–88.

[28] Calcagno V, de Mazancourt C (2010). Glmulti: an R package for easy automated model selection with (generalized) linear models. J Stat Softw.

[29] Yee T. The VGAM package for categorical data analysis. J Stat Softw. 2010;

[30] Fleishman JA, Zuvekas SH (2007). Global self-rated mental health: associations with other mental health measures and with role functioning. Med Care.

[31] Bonikowska A, Helliwell JF, Hou F, Schellenberg G (2013). An assessment of life satisfaction responses on recent statistics Canada surveys. Soc Indic Res.

[32] Mawani FN, Gilmour H (2010). Validation of self-rated mental health. Heal reports.

[33] Ahmad F, Jhajj AK, Stewart DE, Burghardt M, Bierman AS (2014). Single item measures of self-rated mental health: a scoping review. BMC Health Serv Res.

