# Supplementary material for: The fundamental association between mental health and life satisfaction: results from successive waves of a Canadian national survey
Source: BMC Public Health. 2018 Mar 12;18:342. doi: 10.1186/s12889-018-5235-x (PMC5848433; doi:10.1186/s12889-018-5235-x)
Supplement: Supplementary file 1 — Table S1. Life satisfaction mean score and 95% confidence interval for each age category. Table S2. Life satisfaction mean score and 95% confidence interval for each province or territory category. Table S3. Odds ratios of the factors associated with life satisfaction. Table S4. Sensitivity analysis: odds ratios of the factors associated with life satisfaction. (DOCX 44 kb) [file 12889_2018_5235_MOESM1_ESM.docx]

**Additional File 1 to “No health without mental health… and no life satisfaction either: Results from more than 400,000 survey participants.”**

| **Supplementary Table 1: Life satisfaction mean score and 95% confidence interval for each age category.** | | | | |
| --- | --- | --- | --- | --- |
| **Age Category** | **Mean score** | **95% Confidence Interval** | | **p-value** |
| 12-14 | 4.45 | 4.44 | 4.46 | <0.0001 |
| 15-19 | 4.36 | 4.35 | 4.36 | <0.0001 |
| 20-24 | 4.30 | 4.29 | 4.31 | <0.0001 |
| 25-29 | 4.27 | 4.26 | 4.28 | 0.0001 |
| 30-34 | 4.29 | 4.29 | 4.30 | <0.0001 |
| 35-39 | 4.30 | 4.29 | 4.30 | <0.0001 |
| 40-44 | 4.27 | 4.27 | 4.28 | <0.0001 |
| 45-49 | 4.21 | 4.21 | 4.22 | <0.0001 |
| 50-54 | 4.18 | 4.18 | 4.19 | <0.0001 |
| 55-59 | 4.20 | 4.19 | 4.20 | <0.0001 |
| 60-64 | 4.22 | 4.22 | 4.23 | <0.0001 |
| 65-69 | 4.25 | 4.25 | 4.26 | 0.1024 |
| 70-74 | 4.28 | 4.27 | 4.28 | <0.0001 |
| 75-79 | 4.26 | 4.25 | 4.27 | 0.5663 |
| 80 and over | 4.22 | 4.21 | 4.23 | <0.0001 |

| **Supplementary Table 2: Life satisfaction mean score and 95% confidence interval for each province or territory category.** | | | | |
| --- | --- | --- | --- | --- |
| **Province or Territory** | **Mean score** | **95% Confidence Interval** | | **p-value** |
| British Columbia | 4.23 | 4.22 | 4.23 | <0.0001 |
| Northern Territories | 4.24 | 4.23 | 4.25 | 0.0024 |
| Manitoba | 4.24 | 4.24 | 4.25 | 0.0004 |
| Ontario | 4.25 | 4.25 | 4.25 | <0.0001 |
| Alberta | 4.26 | 4.25 | 4.27 | 0.2665 |
| Nova Scotia | 4.26 | 4.25 | 4.27 | 0.6728 |
| Saskatchewan | 4.26 | 4.25 | 4.27 | 0.9035 |
| New Brunswick | 4.27 | 4.26 | 4.28 | 0.0012 |
| Newfoundland and Labrador | 4.30 | 4.29 | 4.31 | <0.0001 |
| Prince Edward Island | 4.32 | 4.30 | 4.33 | <0.0001 |

| **Supplementary Table 3: Odds ratios of the factors associated with life satisfaction (complete table).** | | | | |
| --- | --- | --- | --- | --- |
|  | **Odds ratio** | **95% confidence interval** | | **p-value** |
| **Self-reported mental health** | | | | |
| Poor | Ref |  |  |  |
| Fair | 2.35 | 2.21 | 2.50 | <0.0001 |
| Good | 5.89 | 5.54 | 6.22 | <0.0001 |
| Very good | 11.60 | 10.91 | 12.34 | <0.0001 |
| Excellent | 25.65 | 24.12 | 27.29 | <0.0001 |
| **Self-reported general health** |  |  |  |  |
| Poor | Ref |  |  |  |
| Fair | 2.08 | 1.99 | 2.16 | <0.0001 |
| Good | 3.16 | 3.03 | 3.29 | <0.0001 |
| Very good | 4.92 | 4.73 | 5.13 | <0.0001 |
| Excellent | 7.33 | 7.03 | 7.65 | <0.0001 |
| **Amount of stress in life** |  |  |  |  |
| Extreme | Ref |  |  |  |
| Quite bit | 1.48 | 1.43 | 1.54 | <0.0001 |
| A bit | 2.15 | 2.07 | 2.23 | <0.0001 |
| Not Very | 3.03 | 2.92 | 3.14 | <0.0001 |
| Not at all | 4.68 | 4.50 | 4.87 | <0.0001 |
| **Sense of community belonging** |  |  |  |  |
| Very weak | Ref |  |  |  |
| Some Weak | 1.22 | 1.19 | 1.25 | <0.0001 |
| Some Strong | 1.73 | 1.69 | 1.77 | <0.0001 |
| Very Strong | 2.64 | 2.57 | 2.71 | <0.0001 |
| **Household income** |  |  |  |  |
| Less than 19,999 CAD | Ref |  |  |  |
| Between 20,000 CAD and 39,999 CAD | 1.29 | 1.26 | 1.33 | <0.0001 |
| Between 40,000 CAD and 59,999 CAD | 1.63 | 1.58 | 1.67 | <0.0001 |
| Between 60,000 CAD and 79,999 CAD | 1.96 | 1.90 | 2.01 | <0.0001 |
| More than 80,000 CAD | 2.53 | 2.46 | 2.60 | <0.0001 |
| **Change in health compared to one year ago** |  |  |  |  |
| Much worse | Ref |  |  |  |
| Some worse | 1.38 | 1.31 | 1.46 | <0.0001 |
| The same | 1.71 | 1.62 | 1.81 | <0.0001 |
| Some better | 1.77 | 1.67 | 1.87 | <0.0001 |
| Much better | 2.35 | 2.23 | 2.49 | <0.0001 |
| **Gender** |  |  |  |  |
| Male | Ref |  |  |  |
| Female | 1.27 | 1.26 | 1.29 | <0.0001 |
| **Provinces and Territories** |  |  |  |  |
| Northern Territories | Ref |  |  |  |
| Newfoundland and Labrador | 1.08 | 1.03 | 1.14 | 0.0023 |
| Prince Edward Island | 1.39 | 1.31 | 1.48 | <0.0001 |
| Nova Scotia | 1.30 | 1.24 | 1.36 | <0.0001 |
| New Brunswick | 1.47 | 1.40 | 1.54 | <0.0001 |
| Quebec | 1.25 | 1.20 | 1.31 | <0.0001 |
| Ontario | 1.10 | 1.05 | 1.14 | <0.0001 |
| Manitoba | 1.16 | 1.11 | 1.22 | <0.0001 |
| Saskatchewan | 1.20 | 1.14 | 1.25 | <0.0001 |
| Alberta | 1.09 | 1.05 | 1.14 | <0.0001 |
| British Columbia | 1.06 | 1.02 | 1.11 | 0.0053 |
| **Mood or anxiety disorder** |  |  |  |  |
| Yes | Ref |  |  |  |
| No | 1.08 | 1.05 | 1.10 | <0.0001 |
| **Personal income** |  |  |  |  |
| Between 20,000 CAD and 39,999 CAD | Ref |  |  |  |
| No income | 1.14 | 1.10 | 1.18 | <0.0001 |
| Less than 19,999 CAD | 1.07 | 1.05 | 1.09 | <0.0001 |
| Between 40,000 CAD and 59,999 CAD | 1.01 | 0.99 | 1.03 | 0.311 |
| Between 60,000 CAD and 79,999 CAD | 1.09 | 1.07 | 1.12 | <0.0001 |
| More than 80,000 CAD | 1.20 | 1.17 | 1.23 | <0.0001 |
| **Age category (years old)** |  |  |  |  |
| 40-44 | Ref |  |  |  |
| 15-19 | 1.04 | 1.01 | 1.08 | 0.0152 |
| 20-24 | 1.10 | 1.06 | 1.13 | <0.0001 |
| 25-29 | 1.16 | 1.13 | 1.19 | <0.0001 |
| 30-34 | 1.16 | 1.13 | 1.19 | <0.0001 |
| 35-39 | 1.09 | 1.06 | 1.12 | <0.0001 |
| 45-49 | 1.03 | 1.00 | 1.05 | 0.088 |
| 50-54 | 1.09 | 1.06 | 1.12 | <0.0001 |
| 55-59 | 1.15 | 1.12 | 1.18 | <0.0001 |
| 60-64 | 1.23 | 1.20 | 1.27 | <0.0001 |
| 65-69 | 1.35 | 1.32 | 1.40 | <0.0001 |
| 70-74 | 1.39 | 1.34 | 1.43 | <0.0001 |
| 75-79 | 1.37 | 1.32 | 1.42 | <0.0001 |

| **Supplementary Table 4: Odds ratios of the factors associated with life satisfaction (sensitivity analysis).** | | | | |
| --- | --- | --- | --- | --- |
|  | **Odds ratios** | **95% confidence interval** | | **p-value** |
| **Self-reported mental health** |  |  |  |  |
| Poor | Ref |  |  |  |
| Fair | 2.61 | 2.32 | 2.93 | <0.0001 |
| Good | 6.20 | 5.57 | 6.93 | <0.0001 |
| Very good | 11.21 | 10.02 | 12.56 | <0.0001 |
| Excellent | 20.75 | 18.62 | 23.23 | <0.0001 |
| **Self-reported general health** |  |  |  |  |
| Poor | Ref |  |  |  |
| Fair | 2.24 | 2.08 | 2.42 | <0.0001 |
| Good | 4.18 | 3.88 | 4.51 | <0.0001 |
| Very good | 6.53 | 6.06 | 7.01 | <0.0001 |
| Excellent | 11.27 | 10.41 | 12.19 | <0.0001 |
| **Amount of stress in life** |  |  |  |  |
| Extreme | Ref |  |  |  |
| Quite bit | 1.40 | 1.31 | 1.50 | <0.0001 |
| A bit | 2.09 | 1.95 | 2.23 | <0.0001 |
| Not Very | 2.92 | 2.73 | 3.14 | <0.0001 |
| Not at all | 5.21 | 4.84 | 5.61 | <0.0001 |
| **Sense of community belonging** |  |  |  |  |
| Very weak | Ref |  |  |  |
| Some Weak | 1.14 | 1.09 | 1.19 | <0.0001 |
| Some Strong | 1.68 | 1.61 | 1.76 | <0.0001 |
| Very Strong | 2.57 | 2.45 | 2.69 | <0.0001 |
| **Household income** |  |  |  |  |
| Less than 19,999 CAD | Ref |  |  |  |
| Between 20,000 CAD and 39,999 CAD | 1.14 | 1.09 | 1.18 | <0.0001 |
| Between 40,000 CAD and 59,999 CAD | 1.32 | 1.27 | 1.38 | <0.0001 |
| Between 60,000 CAD and 79,999 CAD | 1.42 | 1.36 | 1.48 | <0.0001 |
| More than 80,000 CAD | 1.68 | 1.61 | 1.75 | <0.0001 |
| **Change in health compared to one year ago** |  |  |  |  |
| Much worse | Ref |  |  |  |
| Some worse | 1.36 | 1.24 | 1.51 | <0.0001 |
| The same | 1.90 | 1.72 | 2.09 | <0.0001 |
| Some better | 1.69 | 1.53 | 1.86 | <0.0001 |
| Much beter | 2.29 | 2.06 | 2.53 | <0.0001 |
| **Gender** |  |  |  |  |
| Male | Ref |  |  |  |
| Female | 1.24 | 1.22 | 1.27 | <0.0001 |
| **Provinces and Territories** |  |  |  |  |
| Northern Territories |  |  |  |  |
| Newfoundland and Labrador | 1.24 | 1.13 | 1.35 | <0.0001 |
| Prince Edward Island | 1.31 | 1.18 | 1.46 | <0.0001 |
| Nova Scotia | 1.30 | 1.20 | 1.42 | <0.0001 |
| New Brunswick | 1.53 | 1.41 | 1.67 | <0.0001 |
| Quebec | 1.25 | 1.17 | 1.34 | <0.0001 |
| Ontario | 1.12 | 1.04 | 1.19 | 0.0016 |
| Manitoba | 1.22 | 1.13 | 1.32 | <0.0001 |
| Saskatchewan | 1.34 | 1.24 | 1.45 | <0.0001 |
| Alberta | 1.11 | 1.03 | 1.20 | 0.0052 |
| British Columbia | 1.06 | 0.99 | 1.14 | 0.1184 |
| **Mood or anxiety disorder** |  |  |  |  |
| Yes | Ref |  |  |  |
| No | 1.12 | 1.08 | 1.16 | <0.0001 |
| **Age category (years old)** |  |  |  |  |
| 45-49 | Ref |  |  |  |
| 12-14 | 1.30 | 1.21 | 1.39 | <0.0001 |
| 15-19 | 1.10 | 1.04 | 1.17 | 0.0008 |
| 20-24 | 1.10 | 1.03 | 1.16 | 0.0025 |
| 25-29 | 1.19 | 1.12 | 1.26 | <0.0001 |
| 30-34 | 1.21 | 1.14 | 1.29 | <0.0001 |
| 35-39 | 1.10 | 1.04 | 1.16 | 0.0015 |
| 40-44 | 1.00 | 0.95 | 1.06 | 0.8784 |
| 50-54 | 1.11 | 1.05 | 1.17 | 0.0003 |
| 55-59 | 1.15 | 1.09 | 1.21 | <0.0001 |
| 60-64 | 1.14 | 1.08 | 1.20 | <0.0001 |
| 65-69 | 1.21 | 1.15 | 1.28 | <0.0001 |
| 70-74 | 1.30 | 1.22 | 1.38 | <0.0001 |
| 75-79 | 1.19 | 1.12 | 1.28 | <0.0001 |
| 80 and over | 1.17 | 1.10 | 1.25 | <0.0001 |
